# Efficacy safety of linaclotide combined with polyethylene glycol for bowel preparation in patients with constipation: a meta-analysis

**DOI:** 10.3389/fgstr.2026.1694950

**Published:** 2026-07-14

**Authors:** Guoli Li, Hongfang Zhang, Lamei Li, Yun Huang

**Affiliations:** Department of Gastroenterology, Qinghai University Affiliated Hospital, Xining, Qinghai, China

**Keywords:** bowel preparation, chronic constipation, linaclotide, meta-analysis, polyethylene glycol, randomized controlled trial

## Abstract

**Background:**

In patients with chronic constipation, standard bowel preparation using polyethylene glycol (PEG) often results in inadequate intestinal cleansing, complicating colonoscopy. This study systematically assessed the efficacy and safety of linaclotide combined with PEG (linaclotide-PEG) compared to PEG alone.

**Methods:**

A systematic search was conducted in PubMed, EMBASE, Web of Science, CENTRAL, CNKI, VIP, and Wanfang databases up to February 1, 2025, to identify randomized controlled trials (RCTs) comparing linaclotide-PEG with PEG monotherapy in constipated patients. Study quality was assessed using the Cochrane Risk of Bias (RoB 2) tool. Data analyses were performed using Stata 16.0.

**Results:**

Eleven RCTs (2022-2024) involving 1,525 participants were included. Compared to PEG alone, linaclotide-PEG significantly improved overall bowel preparation quality [mean difference (MD)=0.82; 95% confidence interval (CI): 0.58-1.05; P < 0.05] and segment-specific cleansing scores (left colon: MD = 0.28; right colon: MD = 0.34; transverse colon: MD = 0.35; all P < 0.05). Additionally, polyp detection rate increased, procedural duration and cecal intubation times decreased, and patients’ willingness to repeat colonoscopy improved significantly (all P < 0.05). The linaclotide-PEG regimen also reduced adverse reaction (nausea and vomiting events) (P < 0.05). No significant publication bias was detected (Egger’s and Begg’s tests: P > 0.05).

**Conclusion:**

Linaclotide-PEG is superior to PEG alone, enhancing bowel cleansing effectiveness, increasing lesion detection rates, shortening procedure times, and reducing adverse events, thus offering clear clinical advantages.

**Systematic review registration:**

https://www.crd.york.ac.uk/PROSPERO/, identifier CRD420250652929.

## Introduction

Colonoscopy is essential for screening, diagnosing, and treating colorectal diseases, with its effectiveness significantly dependent on adequate bowel preparation. Inadequate cleansing approximately triples the risk of missing adenomas ≥5 mm (1) and substantially contributes to failed cecal intubation (2). Poor preparation further prolongs procedure duration, leads to incomplete examinations, increases healthcare costs, and elevates complication risks. Although the European Society of Gastrointestinal Endoscopy recommends achieving adequate preparation in ≥90% of cases, approximately 30% of patients in clinical practice fail to meet this standard (3).

Chronic constipation significantly contributes to inadequate bowel preparation, especially among older adults and women (4). In China, chronic constipation prevalence ranges from 4% to 6% among adults, increasing to 22% in individuals aged >60 years (5). The 2025 American Gastroenterological Association (AGA) consensus identifies constipation as a high-risk factor for poor bowel cleansing. For example, one study reported inadequate bowel preparation in 65.4% of constipated patients, primarily due to delayed colonic transit. Consequently, combining secretagogues, such as linaclotide, with PEG has been recommended to enhance preparation quality in this subgroup.

Common bowel preparation agents include PEG, sulfate-based agents, and phosphate-based agents, with PEG being the most frequently used. Standard PEG regimens typically involve 3-L or 4-L lavage solutions. The 4-L PEG protocol effectively improves bowel cleansing and polyp detection rates. However, its clinical use is often limited by gastrointestinal side effects, including abdominal distension, nausea, and vomiting, reducing patient compliance. Traditional PEG-only regimens frequently fail to provide adequate cleansing for high-risk groups, such as chronic constipation patients, thereby increasing the likelihood of missed lesions. To address these limitations, recent research has focused on combining PEG with adjunctive agents like linaclotide to enhance bowel preparation quality and minimize adverse effects.

Among adjunctive agents, linaclotide, a guanylate cyclase-C (GC-C) agonist, has attracted considerable attention. By binding to GC-C receptors on intestinal epithelial cells, linaclotide elevates intracellular cyclic guanosine monophosphate (cGMP) levels, promoting secretion of chloride and bicarbonate ions, increasing intestinal fluid, and enhancing peristalsis (6). Linaclotide, approved by the United States Food and Drug Administration (FDA), the European Medicines Agency (EMA), and the National Medical Products Administration (NMPA) in China for treating irritable bowel syndrome with constipation (IBS-C), has demonstrated efficacy when combined with 2-L polyethylene glycol (PEG). Notably, studies indicate that this linaclotide-PEG combination achieves bowel preparation quality comparable to conventional 4-L PEG regimens in constipated populations (2).

Despite this promising evidence, there remains no unified consensus regarding the most effective bowel cleansing regimen for constipated individuals undergoing colonoscopy, nor has a comprehensive evaluation of existing research been conducted. Importantly, no systematic meta-analysis has yet assessed the safety and efficacy of linaclotide-PEG combinations in this high-risk population. Therefore, this meta-analysis aims to systematically evaluate the efficacy, measured by key outcomes, and safety, determined by adverse event incidence, of linaclotide-PEG bowel preparation regimens in constipated patients. These findings are expected to provide robust, evidence-based guidance to enhance clinical bowel preparation strategies.

The reporting of this systematic review adheres to the Preferred Reporting Items for Systematic Reviews and Meta-Analyses (PRISMA) 2020 checklist, and the review protocol was registered with the International Prospective Register of Systematic Reviews (PROSPERO) (CRD420250652929).

## Data and methods

### Search strategy

An extensive literature search was conducted across multiple databases, including PubMed, Web of Science, the Cochrane Central Register of Controlled Trials (CENTRAL), EMBASE, CNKI, VIP, and Wanfang, to identify randomized controlled trials (RCTs) comparing bowel cleansing regimens involving linaclotide to conventional PEG-based approaches among constipated patients. The search period spanned from database inception to February 1, 2025. Using the Population, Intervention, Comparator, Outcome (PICO) framework, search terms included Medical Subject Headings (MeSH) and relevant free-text terms such as “Linaclotide,” “Constipation,” “PEG,” and “Bowel Preparation Solutions.” No language or regional restrictions were applied.

Inclusion criteria.

(1) Adult patients with chronic constipation diagnosed based on Rome III or IV criteria; (2) RCTs evaluating linaclotide combined with PEG (linaclotide-PEG) for bowel preparation; (3) Clearly reported outcome measures; (4) Comparable baseline characteristics between intervention and control groups.

Exclusion criteria.

(1) Reviews, case reports, systematic reviews, animal studies, or conference abstracts; (2) RCTs investigating linaclotide without PEG or unrelated to bowel preparation; (3) Incomplete or missing outcome data; (4) Duplicate publications.

### Quality assessment and data extraction

Two reviewers independently performed article selection, methodological quality assessment, and data extraction. Disagreements were resolved by consensus or, if necessary, by consultation with a third reviewer. The Cochrane Risk of Bias 2 (RoB 2) tool was employed to evaluate biases across domains including randomization, allocation concealment, blinding, outcome completeness, selective reporting, and other biases. Studies were classified into three categories: low risk, high risk, or some concerns.

Data extracted included the following:

1. Study characteristics (authors, publication year, design, and sample size);
2. Participant demographics (age, sex, constipation criteria);
3. Intervention details (linaclotide dose, combination regimens);
4. Outcome measures (e.g., bowel cleanliness scores, tolerability, adverse events).

### Statistical analysis

Statistical analyses were conducted using Stata version 16.0. For dichotomous outcomes, relative risks (RRs) with 95% confidence intervals (CIs) were calculated. Continuous outcomes were reported as mean differences (MDs) with 95% CIs. Heterogeneity was assessed using the I^2^ statistic. A fixed-effects model was applied if P > 0.05 and I^2^ < 50%; otherwise, a random-effects model was adopted. For outcomes with significant heterogeneity (I^2^ > 50%), sensitivity analyses verified robustness. Subgroup analyses explored potential sources of heterogeneity, including linaclotide treatment duration, PEG dose (2 L vs. 3–4 L), split-dose versus single-dose administration, and single-center versus multicenter studies. Publication bias was assessed using funnel plots, Egger’s test, and Begg’s test when at least ten studies were available for a specific outcome.

## Results

### Study selection

**Figure 1:**
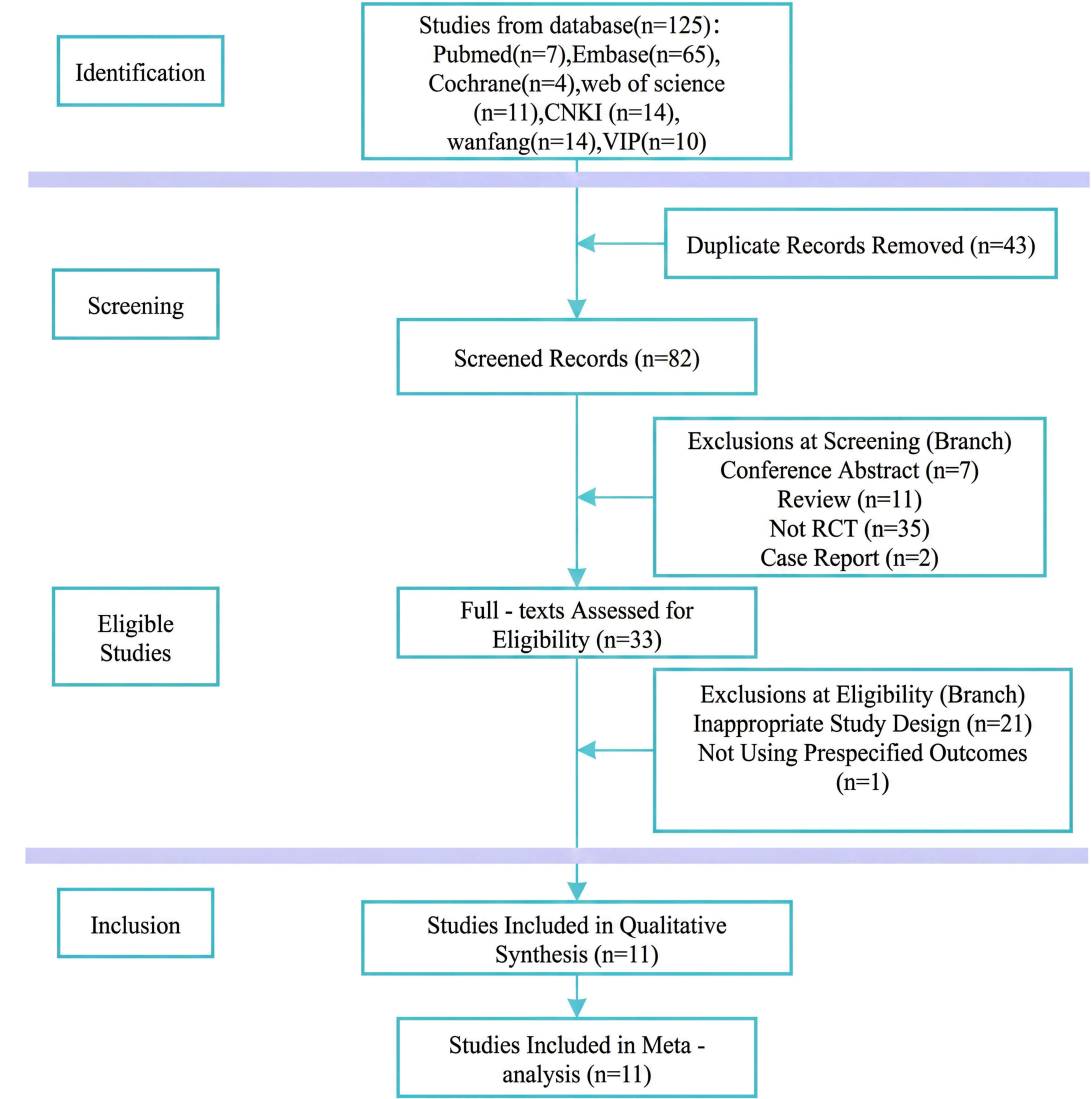
Literature screening flow chart.

Initially, 125 studies were retrieved using specified search terms. After removing 43 duplicates, titles and abstracts of the remaining 82 studies were screened, excluding seven conference abstracts, 11 reviews, two case reports, and 35 non-randomized studies. Twenty-seven articles underwent detailed full-text examination, resulting in the exclusion of an additional 16 studies due to failure to meet inclusion criteria or absence of predetermined outcomes. Ultimately, 11 eligible randomized controlled trials (RCTs) were included in this meta-analysis (2, 6–15), as depicted in the Preferred Reporting Items for Systematic Reviews and Meta-Analyses (PRISMA) flowchart (**Figure 1**).

### Characteristics of included studies

The 11 included RCTs enrolled a total of 1,525 participants: 763 received the linaclotide combined with linaclotide-PEG regimen, and 762 received PEG alone. Both groups showed comparable baseline characteristics. Participants’ ages ranged from 44.5 to 65.2 years. Gender distribution was balanced: 320 males and 443 females in the linaclotide-PEG group, and 321 males and 441 females in the PEG-alone group (**Table 1**).

**Table 1:** Summary overview of the included trials.

Study	Year	Type	Sample size		Treatment		Treatment course	Age (years)		Sex (male/female)		Outcomes
			T	C	T	C		T	C	T	C	
Tingting Chen	2022	single-center	30	30	L+3L-PEG	3L-PEG	3d	44.50 ± 2.35	45.37 ± 2.66	13/17	12/18	① ② ③ ④ ⑥ ⑩
Zhenguang Qi	2022	single-center	58	58	L+3L-PEG	3L-PEG	3d	58.10 ± 13.26	57.72 ± 13.87	27/31	30/28	① ② ③ ④ ⑤ ⑥ ⑨
Jie Zeng	2023	single-center	59	58	L+2L-PEG	3L-PEG	2d	53.6 ± 17.0	55.2 ± 17.3	20/39	21/37	① ② ③ ④ ⑥ ⑧ ⑩
Limin Wu	2023	single-center	50	50	L+3L-PEG	3L-PEG	3d	48.3 ± 10.3	48.9 ± 9.8	24/26	23/27	① ② ③ ④ ⑥ ⑩
Haoxin Xu	2024	single-center	160	159	L+3L-PEG	4L-PEG	3d	53.71 ± 11.92	53.24 ± 11.46	82/78	85/74	① ② ③ ④ ⑤ ⑥ ⑦ ⑧ ⑩
Lianli Wang	2024	multicenter	128	126	L+3L-PEG	4L-PEG	3d	49.03 ± 10.09	49.98 ± 10.42	28/100	22/104	① ② ③ ④ ⑤ ⑥ ⑧ ⑩
Jian Song	2024	single-center	65	64	L+3L-PEG	4L-PEG	6d	49.2 ± 12.6	49.7± 9.4	23/42	25/39	① ② ③ ④ ⑥ ⑧ ⑨ ⑩
Xu Cheng	2024	single-center	50	50	L+3L-PEG	3L-PEG	3d	62.7 ± 7.05	61.1 ± 5.50	26/24	26/24	①⑥
Cunguo Guo	2024	single-center	42	42	L+3L-PEG	3L-EG	3d	65.14 ± 8.09	65.22 ± 8.12	18/24	20/22	① ② ③ ④ ⑤ ⑩ ⑥
Fenglan Huang	2024	single-center	30	31	L+2L-PEG	3L-PEG	2d	45.89 ± 10.21	46.45 ± 10.34	11/19	14/17	① ② ③ ④ ⑤ ⑥ ⑩
Han qing Li	2024	single-center	91	94	L+3L-PEG	3L-PEG	3d	52.62 ± 13.13	54.05 ± 12.02	48/43	43/51	⑥ ⑦ ⑧ ⑩

Outcomes: ① BBPS Total score ② Right side of colon score ③ Mid-colon Score ④ Left colon Score ⑤ Total examination time ⑥ Adverse events ⑦ Adequate preparationrates ⑧ Willingness to repeat the colonoscopy ⑨ Insert time ⑩ Polyp detection rate.

Adverse event:Nausea and Vomiting.

T, treatment group; C, control group.

*The dosage of linaclotide (L) is 290 μg/capsule. The treatment course specifically refers to the oral administration period of linaclotide.

### Methodological quality assessment

**Figure 2:**
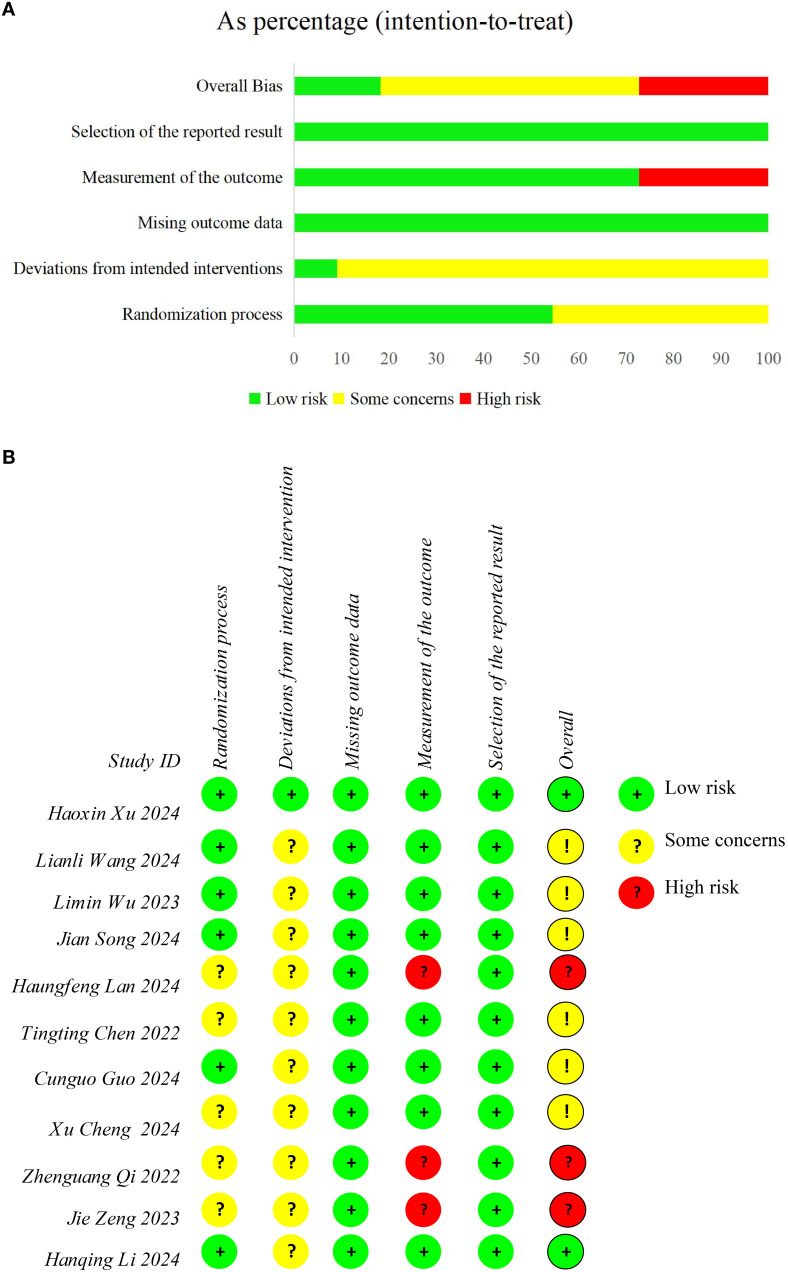
Risk assessment.

All studies were randomized controlled trials. Randomization methods were adequately described in 10 out of 11 studies, except Zeng et al. (6). Blinding procedures were not detailed in three trials (6, 10, 12). All studies reported complete outcome data without evidence of selective reporting. Risk-of-bias assessment outcomes are summarized in **Figure 2**.

**Table 2** presents the summary of findings for the impact of linaclotide combined with PEG on BBPS, total colonoscopy operation time, adequate bowel preparation rate, willingness to repeat colonoscopy, adverse events, and polyp detection rate (**Supplemenyart Appendix 1**).

**Table 2:** GRADE Summary of Findings: Linaclotide Combined with Polyethylene Glycol for Bowel Preparation in Constipated Patients.

Certainty assessment							№ of patients		Effect		Certainty	Importance
№ of studies	Study design	Risk of bias	Inconsistency	Indirectness	Imprecision	Other considerations	PEG+Lin	PEG alone	Relative (95% CI)	Absolute (95% CI)		
BBPS												
102,6-10,12-15	randomised trials	not seriousa	seriousb	not serious	not serious	none	672	668	—	MD 0.82 MD higher (0.58 higher to 1.05 higher)	⨁⨁⨁◯ Moderatea,b	CRITICAL
Total operation time												
52,9,10,12,14	randomised trials	not serious	seriousb	not serious	not serious	none	418	416	—	MD 0 MD (4.79 lower to 1.68 lower)	⨁⨁⨁◯ Moderateb	CRITICAL
Adequate Bowel Preparation Rate												
42,8,11,14	randomised trials	not serious	not serious	not serious	not serious	none	384/429 (89.5%)	311/429 (72.5%)	RR 1.24 (1.16 to 1.32)	174 more per 1,000 (from 116 more to 232 more)	⨁⨁⨁⨁ High	CRITICAL
Willingness to Repeat												
52,6,11,13,14	randomised trials	not serious	not serious	not serious	not serious	none	410/503 (81.5%)	349/501 (69.7%)	RR 1.17 (1.10 to 1.25)	118 more per 1,000 (from 70 more to 174 more)	⨁⨁⨁⨁ High	CRITICAL
Detection Rates of polyp												
82,6,7,9-11,14,15	randomised trials	not serious	not serious	not serious	not serious	none	228/590 (38.6%)	157/591 (26.6%)	RR 1.45 (1.23 to 1.72)	120 more per 1,000 (from 61 more to 191 more)	⨁⨁⨁⨁ High	CRITICAL
Incidence of Adverse Reactions												
112,6-15	randomised trials	not serious	not serious	not serious	not serious	none	148/763 (19.4%)	279/762 (36.6%)	RR 0.53 (0.45 to 0.62)	172 fewer per 1,000 (from 201 fewer to 139 fewer)	⨁⨁⨁⨁ High	CRITICAL

CI, confidence interval; MD, mean difference; RR, risk ratio.

a, Although two included trials were rated as high risk of bias, these studies did not dominate the pooled effect estimate. Furthermore, the direction of the effect was consistent across trials with low, some concerns, and high risk of bias. Therefore, no downgrading for risk of bias was applied.

b, Rated down 1 level for inconsistency because significant heterogeneity was observed across the included trials (I² > 80%).

### Meta-analysis

#### Bowel cleanliness

**Figure 3:**
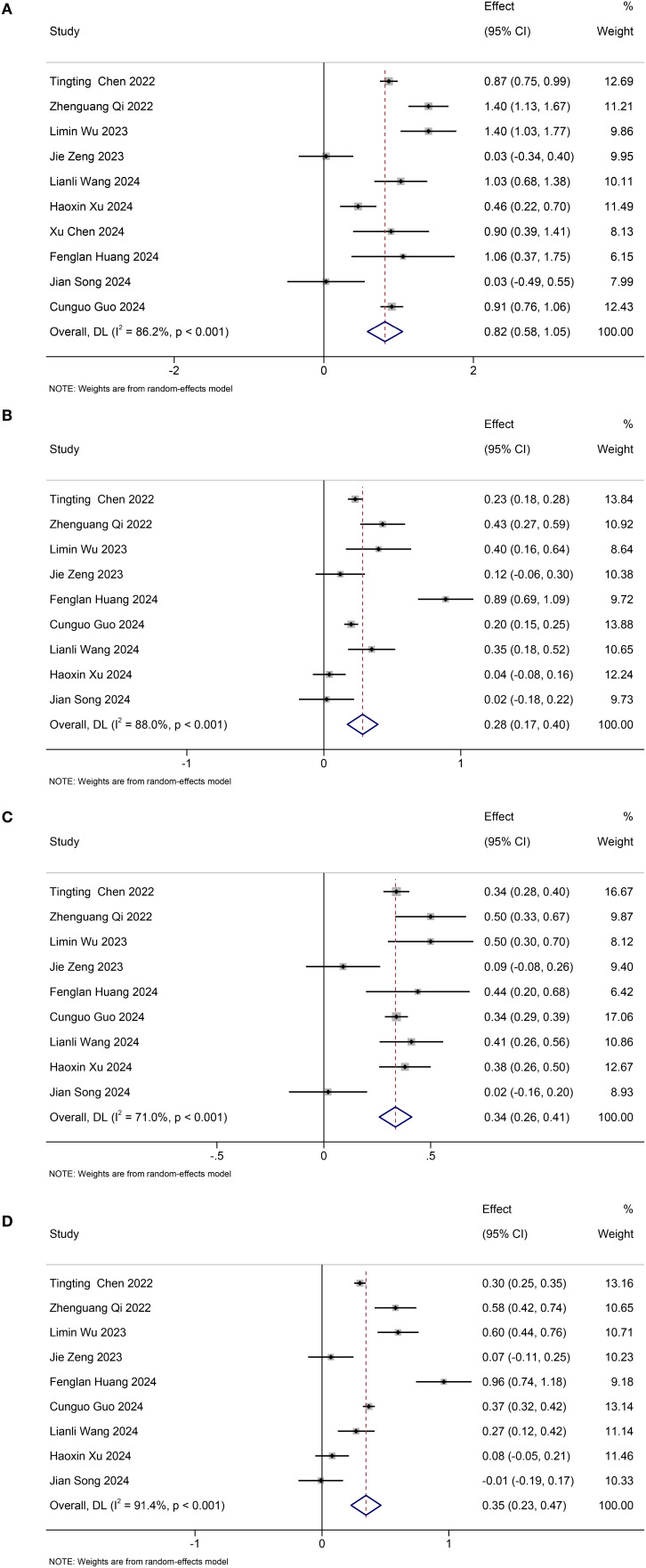
Forest plot of BBPS. **(A)** Total Score; **(B)** Left colon Score; **(C)** Right colon Score; **(D)** Mid-colon Score.

Ten studies involving 1,340 patients (linaclotide-PEG group: n = 672; PEG-alone group: n = 668) compared total Boston Bowel Preparation Scale (BBPS) scores. Due to substantial heterogeneity (I² = 86.2%, P < 0.05), a random-effects model was utilized. Linaclotide-PEG significantly improved bowel cleansing compared to PEG alone, as indicated by total BBPS scores (MD = 0.82; 95% CI: 0.58–1.05; P < 0.05; **Figure 3A**). Nine studies also assessed BBPS scores for colonic segments, including 622 patients in the linaclotide-PEG group and 618 in the PEG-alone group. Moderate to substantial heterogeneity was present for each segment (I² > 50%, P < 0.001); thus, a random-effects model was adopted. Linaclotide-PEG significantly improved cleanliness in the left colon (MD = 0.28; 95% CI: 0.17–0.40; P < 0.05), right colon (MD = 0.34; 95% CI: 0.26–0.41; P < 0.05), and transverse colon (MD = 0.35; 95% CI: 0.23–0.47; P < 0.05) (**Figures 3B–D**).

#### Total colonoscopy operation time

**Figure 4:**
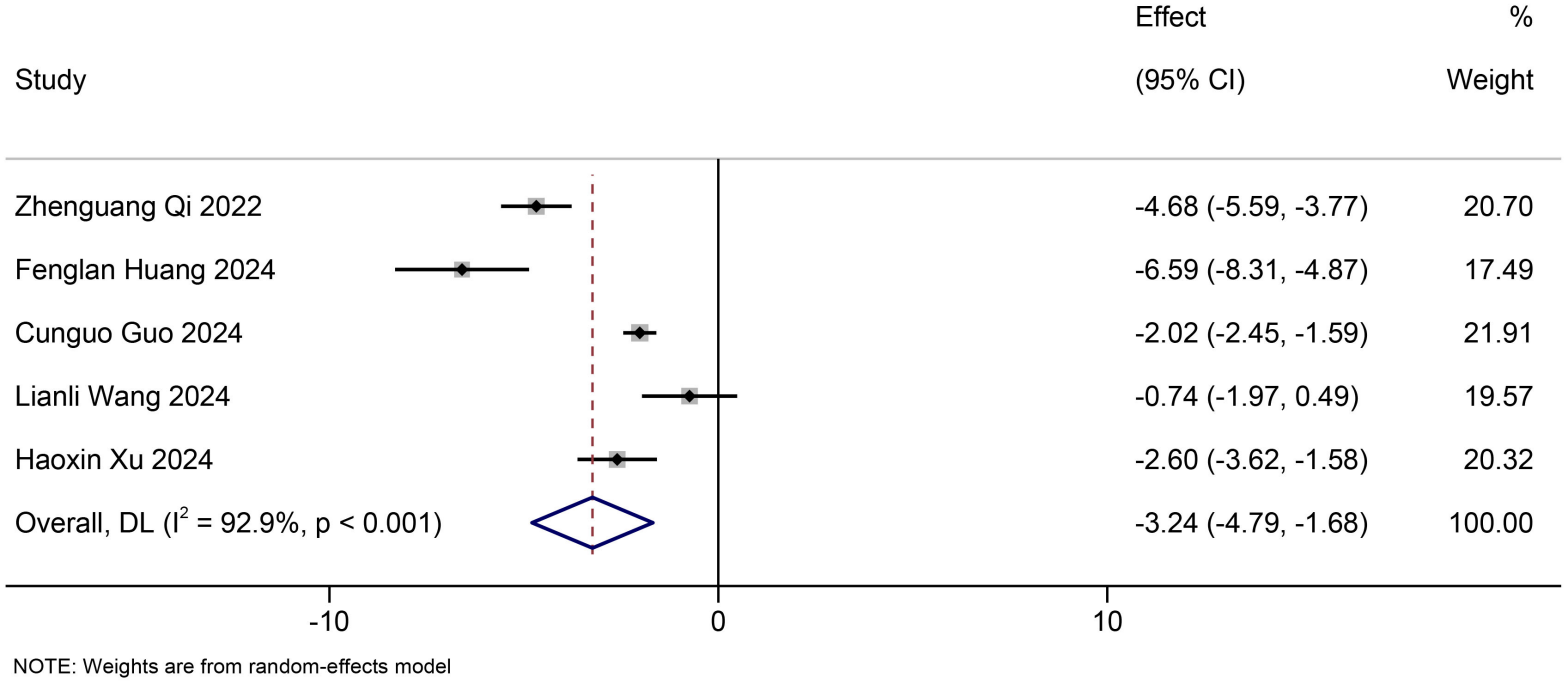
Forest plot of total examination time.

Five studies involving 834 patients (linaclotide-PEG: n = 418; PEG-alone: n = 416) evaluated total colonoscopy duration. Significant heterogeneity (I^2^ = 92.9%, P < 0.05) justified a random-effects model. The linaclotide-PEG group demonstrated significantly shorter procedure durations than PEG alone (MD = -3.24 min; 95% CI: -4.79 to -1.68; P < 0.05; **Figure 4**).

#### Iintubation time

**Figure 5:**
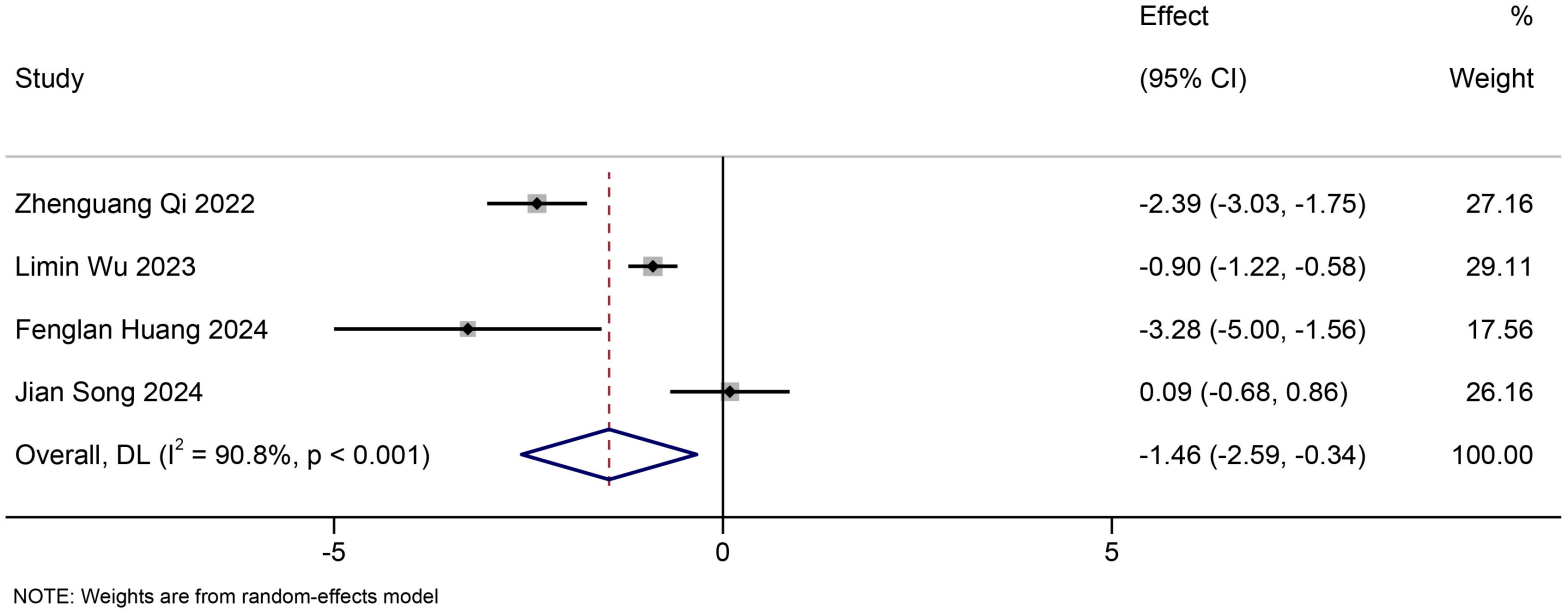
Forest plot of insert time.

Four studies evaluated colonoscopy insertion time, involving 406 patients (linaclotide-PEG: n = 203; PEG-alone: n = 203). Due to significant heterogeneity (I^2^ = 90.8%, P < 0.05), a random-effects model was used. The linaclotide-PEG group showed significantly shorter insertion times compared with PEG alone (MD = -1.46 min; 95% CI: -2.59 to -0.34; P < 0.05; **Figure 5**).

#### Adequate bowel preparation rate

**Figure 6:**
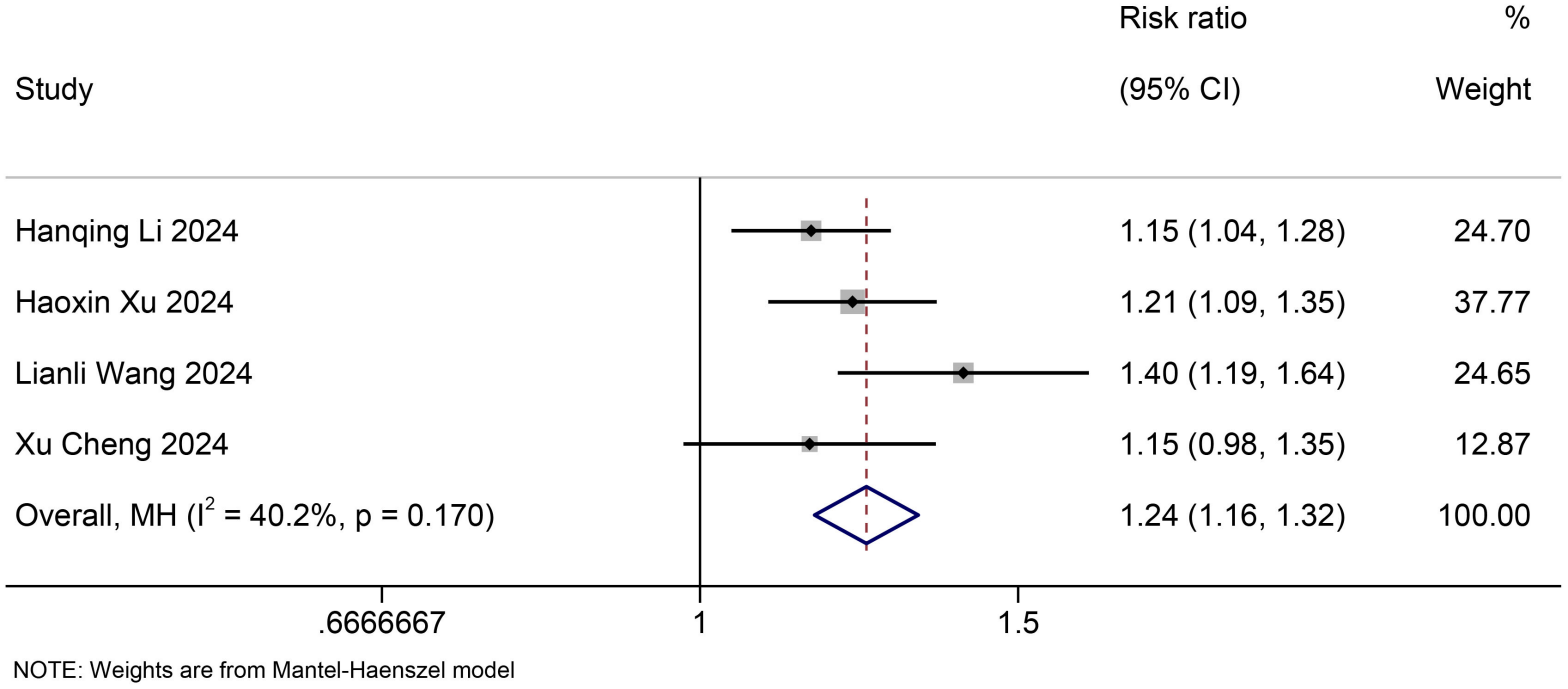
Forest plot of adequate preparationrates.

Four studies assessed adequate bowel preparation rates, involving 858 patients (linaclotide-PEG: n = 429; PEG-alone: n = 429). With low heterogeneity (I² = 40.2%, P = 0.17), a fixed-effects model was applied. Linaclotide-PEG significantly improved adequate preparation rates versus PEG alone (relative risk [RR] = 1.24; 95% CI: 1.16–1.32; P < 0.05), representing a 24% relative increase (**Figure 6**). The pooled risk difference (RD) showed that combination therapy increased adequate bowel preparation by 17.1% (RD = 0.171, 95% CI: 0.121-0.222; p < 0.05), with low heterogeneity across studies (I² = 9.8%).

#### Willingness to repeat

**Figure 7:**
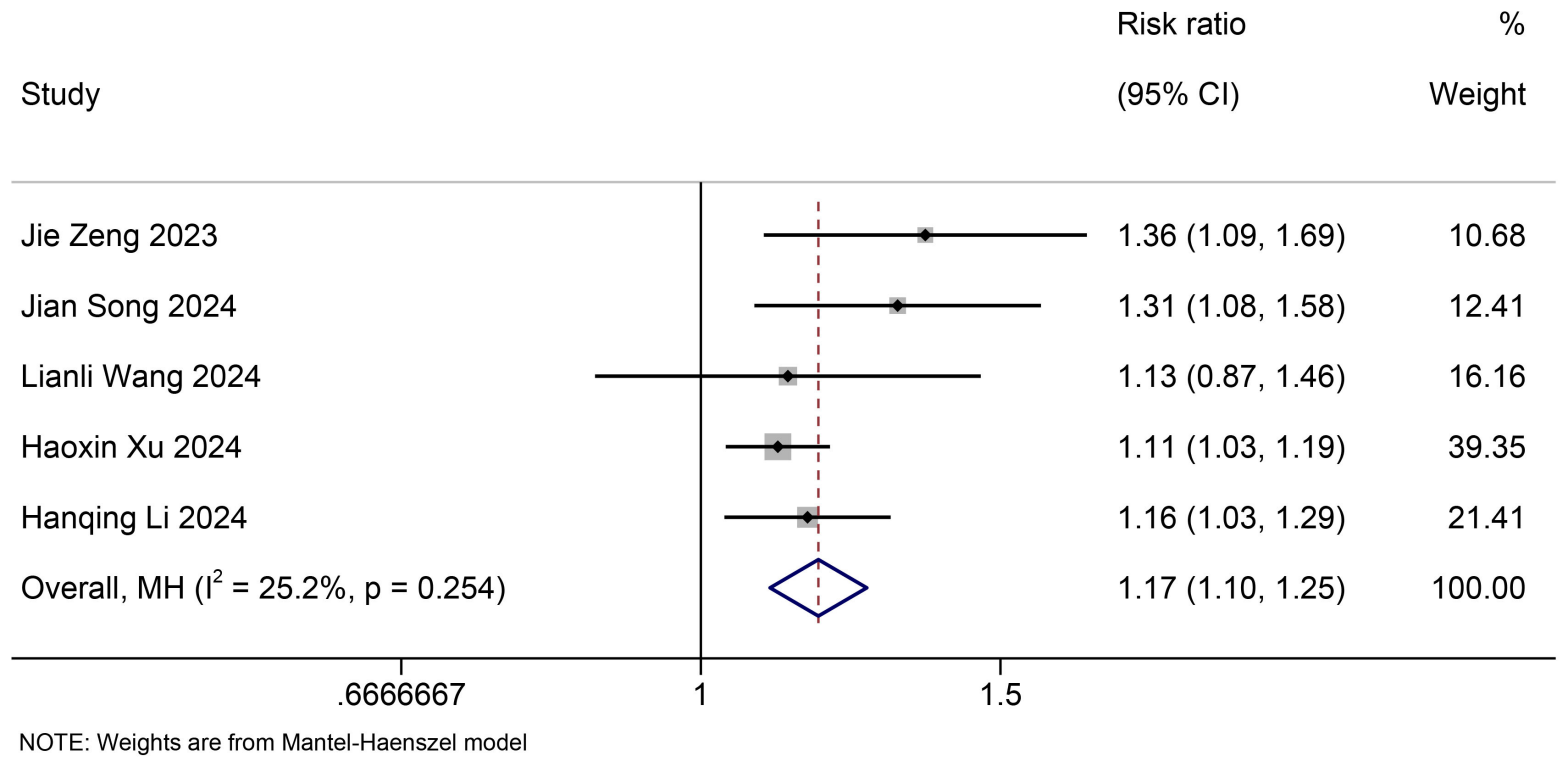
Forest plot of willingness to repeat the colonoscopy.

Five studies evaluated willingness-to-repeat colonoscopy rates, involving 759 patients (linaclotide-PEG: n = 410; PEG-alone: n = 349). Low heterogeneity (I² = 25.2%, P = 0.25) supported a fixed-effects model.Linaclotide-PEG significantly increased willingness-to-repeat rates compared to PEG alone (RR = 1.17; 95% CI: 1.10–1.25; P < 0.05), indicating a 17% relative increase (**Figure 7**).

#### Detection rates of polyp

**Figure 8:**
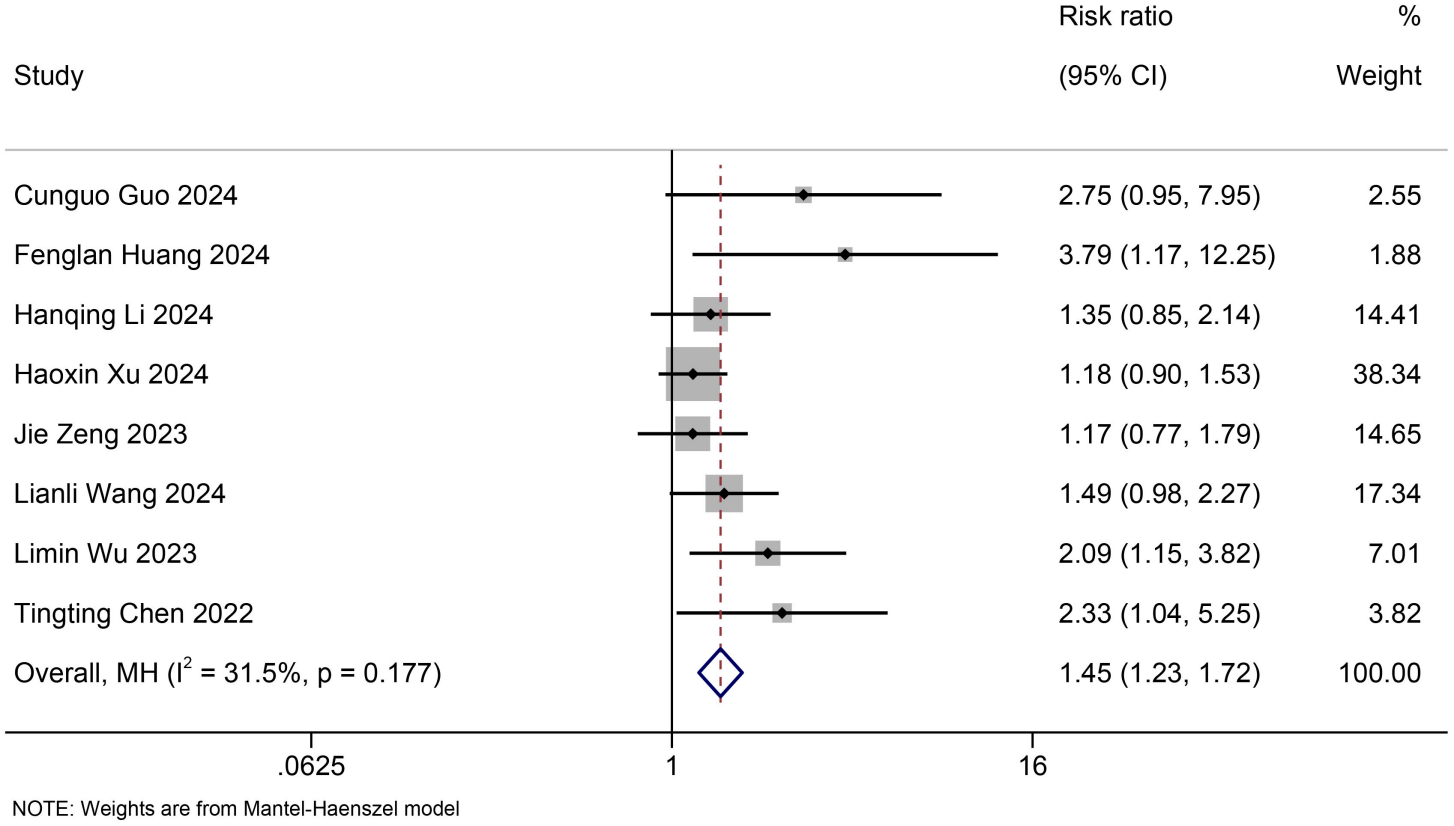
Forest plot of polyp detection rate.

Eight trials compared detection rates of polyps between linaclotide-PEG (n = 590) and PEG-alone (n =591) groups. Moderate heterogeneity (I² = 31.5%, P = 0.17) necessitated a random-effects model. Linaclotide-PEG significantly increased lesion detection rates (RR = 1.45; 95% CI: 1.23–1.72; P < 0.05; **Figure 8**).

#### Incidence of adverse reactions

**Figure 9:**
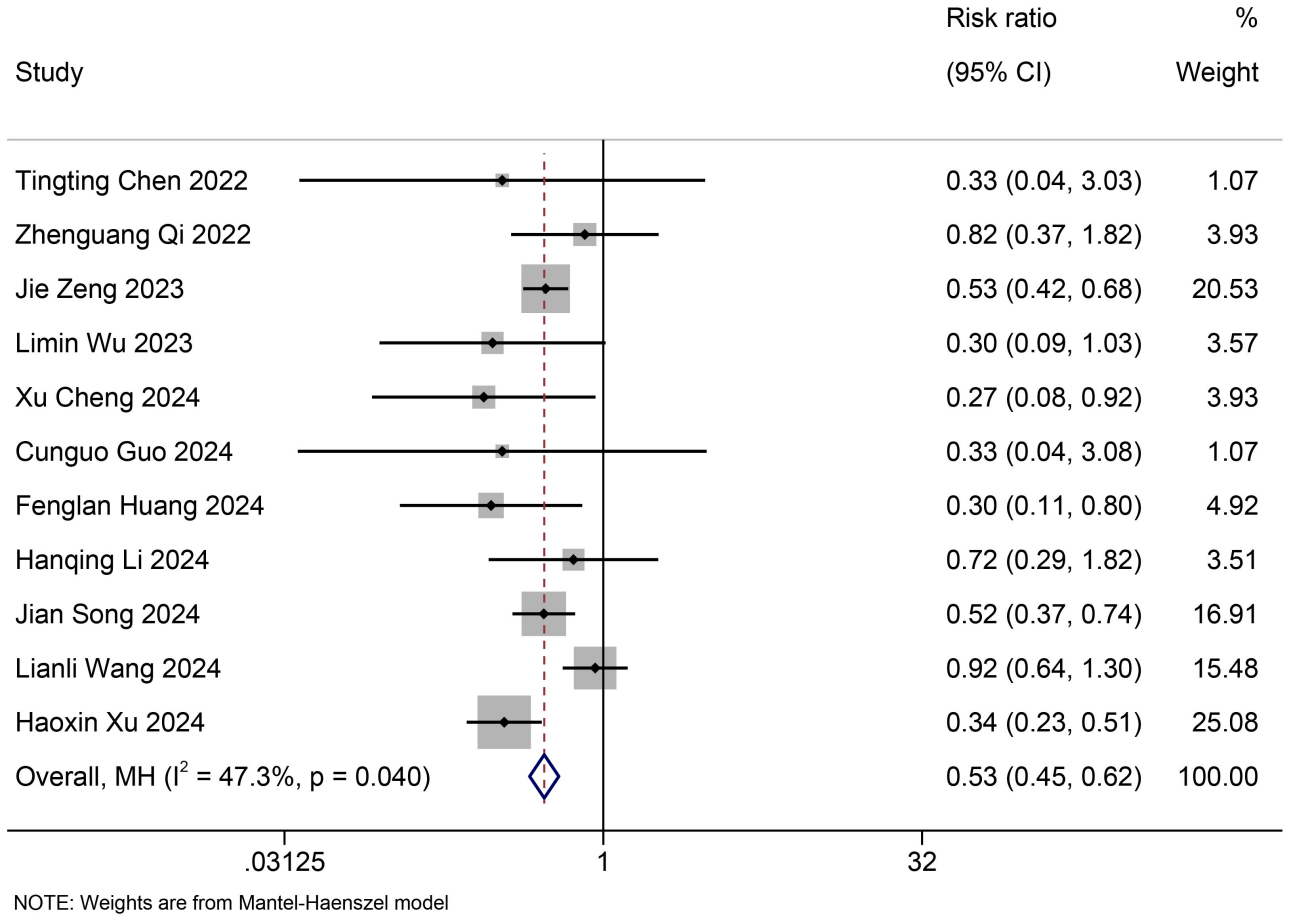
Forest plot of adverse events.

Eleven studies compared adverse reaction rates between linaclotide-PEG (n = 763) and PEG-alone (n = 762) groups. Due to moderate heterogeneity (I^2^ = 47.3%, P = 0.04), a random-effects model was applied. Linaclotide-PEG significantly reduced adverse reaction rates compared with PEG alone (RR = 0.53; 95% CI: 0.45–0.62; P < 0.05; **Figure 9**).

To explore subgroup effects on the association between regimen characteristics and BBPS scores, prespecified subgroup analyses were conducted. Significant variations were observed according to linaclotide treatment duration (6-day MD = 0.03, 95% CI: −0.49–0.55, I² = 0% vs. 2-day MD = 0.50, 95% CI: −0.50–1.51 vs. 3-day MD = 0.98, 95% CI: 0.77–1.19, P < 0.05) and study design (multicenter MD = 1.03, 95% CI: 0.68–1.38 vs. single-center MD = 0.79, 95% CI: 0.54–1.05, P < 0.05). For other stratification factors (PEG dose and split- vs. single-dose administration), results remained largely consistent (**Supplementary Appendix 2**). Sensitivity analyses confirmed stable effect sizes for total and segmental BBPS scores across studies, suggesting reliable meta-analysis findings (**Supplementary Appendix 3**). Publication bias evaluation for total BBPS scores revealed no significant bias (P = 0.72), with the corresponding funnel plot provided (**Supplementary Appendix 4**).

## Discussion

Constipation is a common gastrointestinal disorder worldwide. Epidemiological data indicate that the prevalence among adults is approximately 14%-16%, with higher rates observed in women and elderly individuals, and it increases with age (4, 16). In China, constipation prevalence ranges from 3% to 11% in the general population, rising to 13%–32.6% among older adults, and approximately 17.6% in individuals over 65 years, of which functional constipation accounts for more than 60% (17). In constipated patients, slow intestinal transit and dry, hard stools not only reduce quality of life but also complicate bowel preparation. The incidence of inadequate bowel cleansing in these patients is two to three times higher than in non-constipated populations (18), directly impacting the quality of endoscopic examinations and disease detection rates, which may have further implications for patient prognosis. Despite the availability of optional medications proposed in relevant consensuses and guidelines, the search for effective treatments to address unmet clinical needs continues.

Currently, osmotic laxatives represent the primary choice for bowel preparation, with split-dose oral administration of 3–4 L PEG as the recommended first-line regimen. However, single-agent PEG administration has significant limitations in constipated patients. Due to the large volume required, approximately 5%–15% of patients fail to complete the preparation (19), and about 30%–40% achieve only suboptimal cleansing (BBPS score < 6) because of insufficient intestinal motility. Additionally, PEG commonly causes discomfort such as abdominal distension and nausea, resulting in compliance rates of only 60%–70%, with some patients discontinuing treatment due to intolerance (20). These data suggest that patients favor low-volume PEG (<4 L) regimens due to better tolerability, whereas physicians prioritizing bowel preparation quality may continue to prefer high-volume PEG-based protocols. This divergence represents an ongoing clinical challenge. In addition to PEG, alternative bowel cleansing agents—such as magnesium sulfate, sodium phosphate, and oral sulfate solutions—may be selected based on individual patient characteristics. These agents can also be combined at low doses with adjuncts, including ascorbic acid or citrate esters, to enhance cleansing efficacy. Importantly, the 2025 USMSTF(US Multi-Society Task Force) guidelines indicate that split-dose low-volume PEG combined with other bowel-cleansing agents can achieve efficacy comparable to that of traditional high-volume PEG regimens (21).

The 2023 Chinese colonoscopy guidelines indicate that for patients with risk factors for inadequate bowel preparation, combination therapy can be considered to improve bowel cleansing efficacy (5). Commonly selected agents include linaclotide, lactulose, and other similar medications. As a secretagogue, linaclotide has a targeted laxative mechanism. It activates guanylate cyclase-C receptors on intestinal epithelial cells, promotes the production of cGMP, increases intestinal fluid secretion, accelerates intestinal peristalsis, and reduces visceral hypersensitivity (6). These pharmacological properties render linaclotide particularly suitable for patients with functional constipation, especially those with impaired intestinal motility or inadequate responses to conventional laxatives. Linaclotide significantly increases the weekly frequency of spontaneous bowel movements, improves stool consistency, and exhibits favorable safety, thereby providing a theoretical basis for its combination with PEG in bowel preparation among constipated patients.°Clinical studies have demonstrated that in patients with constipation, combining linaclotide with 2 L PEG significantly increases BBPS scores compared to 2 L PEG alone, while no significant difference is observed relative to 4 L PEG (22). Furthermore, Stein et al. reported that linaclotide combined with 3 L PEG improves right-colon bowel preparation quality, shortens the time to first bowel movement, and enhances patient satisfaction and comfort compared with 3 L PEG alone (23). A recent meta-analysis evaluating different linaclotide dosages combined with PEG found that 3 L PEG + 3linaclotide significantly increases adenoma detection rates compared with PEG alone, whereas 2 L PEG + 2 linaclotide provides superior patient tolerability (24). Collectively, these findings underscore the clinical value of linaclotide–PEG combination regimens.

In this study, we specifically focus on patients with chronic constipation, a population at elevated risk of inadequate bowel preparation despite clinical indications for colonoscopy. Through systematic keyword-based screening, 11 relevant articles were identified and included. The results indicate that for patients with chronic constipation, the linaclotide combined with PEG bowel preparation regimen was significantly superior to PEG alone in terms of cleanliness of the entire colon and individual colonic segments, as well as a significantly higher rate of adequate bowel preparation. The pooled risk difference of 17.1% indicates that approximately 17 additional patients per 100 treated would achieve adequate bowel preparation with the combination regimen, corresponding to a number needed to treat (NNT) of approximately 6. Given the high baseline risk of inadequate bowel preparation in patients with constipation, this represents a clinically meaningful benefit.

Moreover, improved bowel cleanliness led to higher detection rates of polyps in the linaclotide–PEG group. Regarding procedural efficiency, total procedure duration was shorter and cecal intubation faster in the linaclotide–PEG group, thus enhancing ov erall endoscopic examination efficiency. Importantly, detection rate only reflected endoscopic lesion identification, independent of subsequent cold/hot snare polypectomy or EMR, and was not influenced by resection methods. Given such inconsistencies, this finding is hypothesis-generating and merely suggests a potential increase in detection rate, rather than a definitive conclusion. Concerning safety, the incidence of gastrointestinal adverse events(nausea and vomiting) was lower with the combined regimen, suggesting better safety and tolerability alongside improved cleansing efficacy and operational efficiency. Although diarrhea was reported in several studies, only one detailed its severity, confirming mild symptoms without treatment discontinuation. However, most trials did not systematically document diarrhea severity, electrolyte disturbances, or patient withdrawals due to adverse events, limiting comprehensive quantitative safety evaluation.

Subgroup analyses suggested that study setting (single-center vs multicenter) and linaclotide dose may contribute to between-study heterogeneity. In contrast, PEG volume and dosing regimen did not appear to significantly influence the pooled estimates. Further well-designed multicenter studies using standardized linaclotide dosing protocols are needed to reduce heterogeneity and enhance the generalizability of the findings.

In clinical practice, regimen selection should balance cleansing efficacy, patient tolerability, and potential effects on lesion detection. The combination of 2 L PEG plus linaclotide provides adequate bowel preparation with improved tolerability and is a reasonable choice for constipated patients intolerant of large-volume regimens. Conversely, higher-volume PEG (3–4 L) combined with linaclotide may further enhance bowel cleansing and potentially improve lesion detection, though such findings should be interpreted cautiously. This regimen might be beneficial for patients at higher risk of inadequate preparation or when maximal mucosal visualization is essential. Standard 4 L PEG remains a viable alternative when combination therapies are unavailable. Ultimately, regimen choice should be individualized based on patient characteristics and clinical priorities.

In summary, the linaclotide–PEG regimen demonstrated comprehensive advantages in bowel preparation for constipated patients. Its bowel-cleansing efficacy significantly exceeded that of PEG alone by providing a clearer endoscopic field, shortening procedural time, facilitating smoother cecal intubation, and thereby improving physician efficiency. Additionally, this regimen increased the detection rates of clinically significant lesions, such as intestinal polyps, thus aiding in early lesion identification. From a safety perspective, linaclotide combined with PEG reduced gastrointestinal adverse events and improved patient compliance, highlighting its role as a preferred bowel preparation strategy with balanced efficacy and safety for patients with constipation.

## Shortcomings and limitations

This study had several limitations. First, all included trials were conducted in China; therefore, regional differences in constipation prevalence, dietary habits, and clinical practices might restrict the generalizability (external validity) of findings to non-Chinese populations. Second, several included RCTs inadequately reported blinding or allocation concealment, potentially affecting the accuracy of risk-of-bias assessments. Future studies should conduct pragmatic trials among diverse ethnic groups and populations to address these limitations and further evaluate the efficacy and safety of linaclotide combined with PEG regimens, particularly within common Western bowel preparation protocols such as split-dose 2 L PEG-ascorbic acid and sulfate-based preparations. Third, PEG administration methods varied among trials (e.g., split-dose vs. single-dose regimens). Additionally, BBPS scores are inherently subjective and can be influenced by evaluator bias in non-blinded studies, leading to heterogeneity that may underestimate the true bowel-cleansing efficacy of the linaclotide–PEG regimen.

## Data Availability

The original contributions presented in the study are included in the article/**Supplementary Material**. Further inquiries can be directed to the corresponding author.

## References

[1] Clark BT Protiva P Nagar A Imaeda A Ciarleglio MM Deng Y . Quantification of adequate bowel preparation for screening or surveillance colonoscopy in men. Gastroenterology. (2016) 150(2):396–405. doi: 10.1053/j.gastro.2015.09.041 26439436 PMC4728019

[2] Xu H He Z Liu Y Li Z Wang Y . Application of linaclotide in bowel preparation for colonoscopy in patients with constipation: a prospective randomized controlled study. J Gastroenterol Hepatol. (2024) 39(12):2752–9. doi: 10.1111/jgh.16734 39252470

[3] Liu WQ Shu L Zhou X Wang XF Liu S Shi ZH . Evaluation of the efficacy of polyethylene glycol in combination with different doses of linaclotide in a fractionated bowel preparation for colonoscopy: a prospective randomized controlled study. Int J Colorectal Dis. (2024) 39(1):143. doi: 10.1007/s00384-024-04718-4 39289199 PMC11408392

[4] Agarwal P Jha BK Somagoni J B SS Modh V Chakilam SK . Efficacy and safety of elobixibat in patients with chronic constipation—a randomized, multicenter, double-blind, placebo-controlled, parallel-group study from India. Indian J Gastroenterol. (2025) 44(3):336–44. doi: 10.1007/s12664-024-01719-7 39985701 PMC12141165

[5] Colorectal Group, Digestive Endoscopy Branch of Chinese Medical Association . Expert consensus statement on bowel preparation for colonoscopy (2023, Guangzhou). Chin J Digest Endosc. (2023) 40(6):421–30. doi: 10.3760/cma.j.cn321463-20230607-00230

[6] Zeng J Gu S . Effect of linaclotide combined with PEG on bowel preparation before colonoscopy in constipated patients. Mod Med Health. (2023) 39. doi: 10.3389/fmed.2026.1686654 41658593 PMC12875982

[7] Chen T Luo Z He J Li X Yang F . Effect of linaclotide combined with compound polyethylene glycol in bowel preparation for colonoscopy in constipated patients. Chin Mod Doct. (2022) 60:71–5.

[8] Cheng X Wei S Dong Y . Effect of linaclotide combined with compound polyethylene glycol electrolyte powder in intestinal cleansing in patients with chronic constipation. Clin Med Res Pract. (2024) 9:45–8.

[9] Guo C Fu S . Evaluation of the effect of linaclotide combined with compound polyethylene glycol electrolyte in bowel preparation for elderly patients with chronic constipation. Chin Med Herald. (2024) 21:95–7.

[10] Huang F Long J Luo T Zhu J Yang Y . Clinical study on linaclotide combined with compound polyethylene glycol electrolyte powder in bowel preparation for colonoscopy in constipated patients. Med Theory Pract. (2024) 37(9):1576–1578.

[11] Li H Li Y Qi X Zhang P Yu Y Tian Z . Study on efficacy and safety of linaclotide combined with polyethylene glycol in bowel preparation for patients with functional constipation. Chin J Digest. (2024) 44(9):605–612. doi: 10.3760/cma.j.cn311367-20240131-00046

[12] Qi Z Li X Zhang W . Clinical application of linaclotide combined with compound polyethylene glycol electrolyte powder in bowel preparation for patients with chronic constipation. J Kunming Med Univ. (2022) 43:130–5.

[13] Song J Xu Y Chen C Qi X Hu P Ying X . The effects of combined use of linaclotide and polyethylene glycol electrolyte powder in colonoscopy preparation for patients with chronic constipation. Surg Laparosc Endosc Percutan Tech. (2024) 34(2):129–135. doi: 10.1097/SLE.0000000000001273 38444073

[14] Wang L Zhang Y Li J Ran Y Wang X Ma X . Efficacy of polyethylene glycol electrolyte powder combined with linaclotide for colon cleansing in patients with chronic constipation undergoing colonoscopy: a multicenter, single-blinded, randomized controlled trial. Clin Transl Gastroenterol. (2024) 15(6):e1. doi: 10.14309/ctg.0000000000000708 38713137 PMC11196075

[15] Wu L-M Xu C-L Xia X-P Wang Y . Linaclotide combined with polyethylene glycol regimen for bowel preparation in patients with chronic constipation: a prospective randomized controlled study. World Chin J Digestol. (2023) 31(19):816–21. doi: 10.11569/wcjd.v31.i19.816

[16] Yang L Zong Y Meng F Liu Y Liu W . Comparative efficacy and safety of lubiprostone and osmotic laxatives in chronic idiopathic constipation: a systematic review and network meta-analysis. J Gastroenterol Hepatol. (2025) 40(2):387–97. doi: 10.1111/jgh.16844 39660667

[17] Du X Liu S Jia P Wang X Gan J Hu W . Epidemiology of constipation in elderly people in parts of China: a multicenter study. Front Public Health. (2022) 10:823987. doi: 10.3389/fpubh.2022.823987 35784241 PMC9240593

[18] Park HJ Chae MH Kim HS Kim JW Kim MY Baik SK . Colon transit time may predict inadequate bowel preparation in patients with chronic constipation. Intest Res. (2015) 13(4):339–45. doi: 10.5217/ir.2015.13.4.339 26576140 PMC4641861

[19] Li Z Linghu E . Concise version of guidelines for bowel preparation related to digestive endoscopy diagnosis and treatment in China (2019, Shanghai). Chin J Digest. (2019) 39:438–43.

[20] Ness RM Manam R Hoen H Chalasani N . A predictive model identifies patients likely to have inadequate bowel preparation for colonoscopy. Am J Gastroenterol. (2012) 107:735–42. doi: 10.1016/j.cgh.2011.12.037

[21] Jacobson BC Anderson JC Burke CA Dominitz JA Gross SA May FP . Optimizing bowel preparation quality for colonoscopy: consensus recommendations by the US Multi-Society Task Force on Colorectal Cancer. Am J Gastroenterol. (2025) 120(4):738–64. doi: 10.14309/ajg.0000000000003287 40035345

[22] Zhang M Zou W Xu C Jia R Liu K Xu Q . Polyethylene glycol combined with linaclotide is an effective and well-tolerated bowel preparation regimen for colonoscopy: an endoscopist-blinded, randomized, controlled trial. Eur J Gastroenterol Hepatol. (2021) 33(1S Suppl 1):e625–e633. doi: 10.1097/MEG.0000000000002184 34034273

[23] Stein DJ Copland A McDaniel D Rao S . Single-dose linaclotide is equal in efficacy to polyethylene glycol for bowel preparation prior to capsule endoscopy. Dig Dis. (2019) 37(4):297–302. doi: 10.1159/000496350 30731474

[24] Elgendy MS Rajab I Najah Q Faheem MA Elsawy OK Taha HI . Combined linaclotide and polyethylene glycol electrolyte for colonoscopy preparation: a network meta-analysis of 14 randomized controlled trials. Int J Colorectal Dis. (2025) 40(1):143. doi: 10.1007/s00384-025-04931-9 40528061 PMC12174239

