# Supplementary material for: Efficacy safety of linaclotide combined with polyethylene glycol for bowel preparation in patients with constipation: a meta-analysis
Source: Front Gastroenterol (Lausanne). 2026 Jul 14;5:1694950. doi: 10.3389/fgstr.2026.1694950 (PMC13407309; doi:10.3389/fgstr.2026.1694950)
Supplement: Supplementary file 1 [file DataSheet1.docx]

Table II GRADE Summary of Findings: Linaclotide Combined with Polyethylene Glycol for Bowel Preparation in Constipated Patients


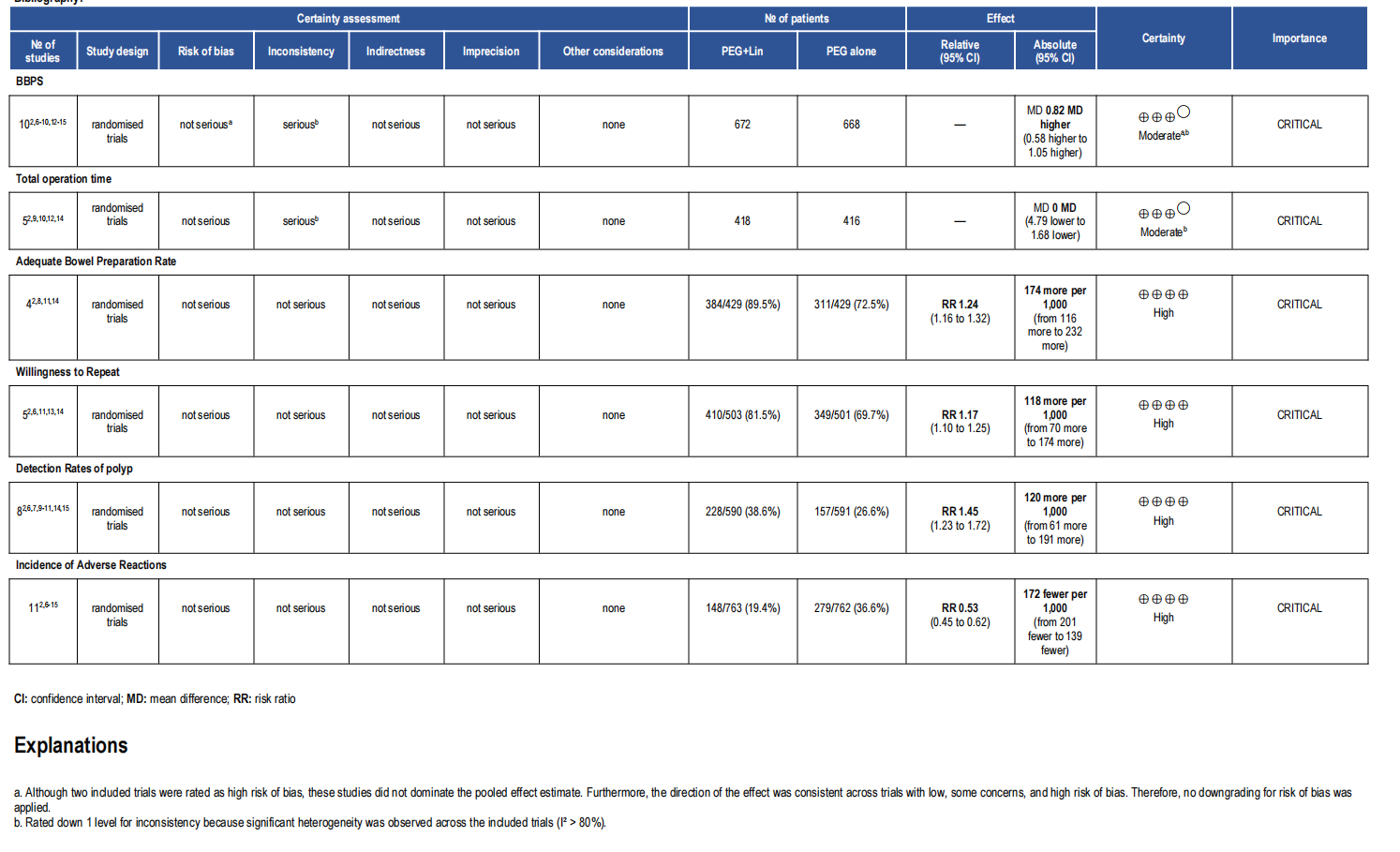


Subgroup analysis forest plot

| 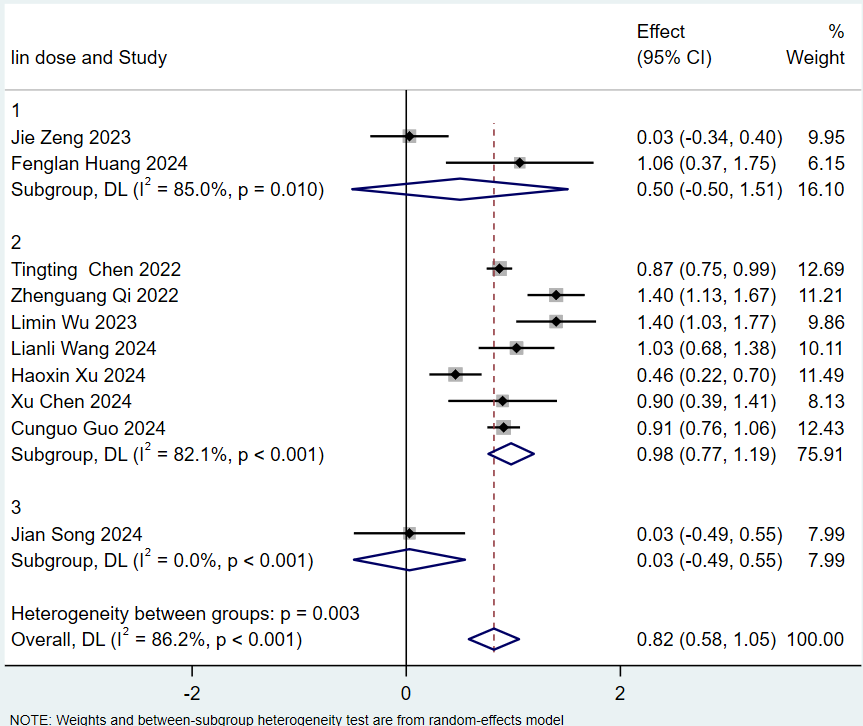  A | 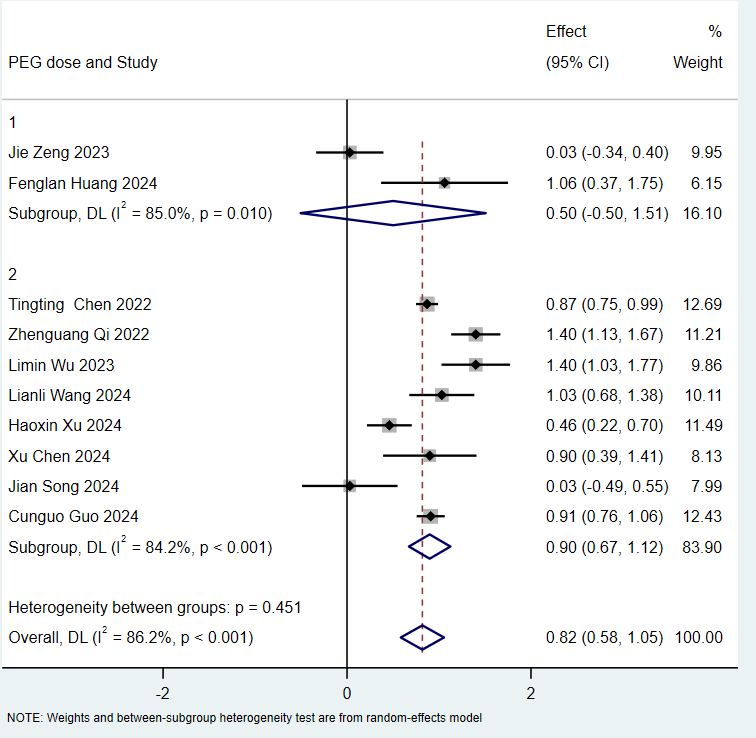  B |
| --- | --- |
| 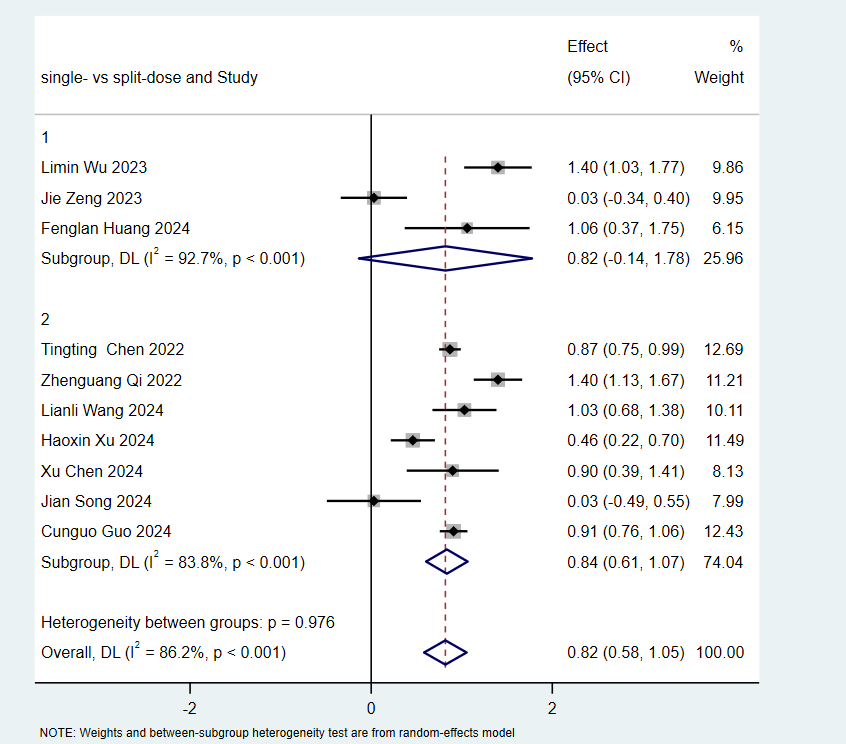  C | 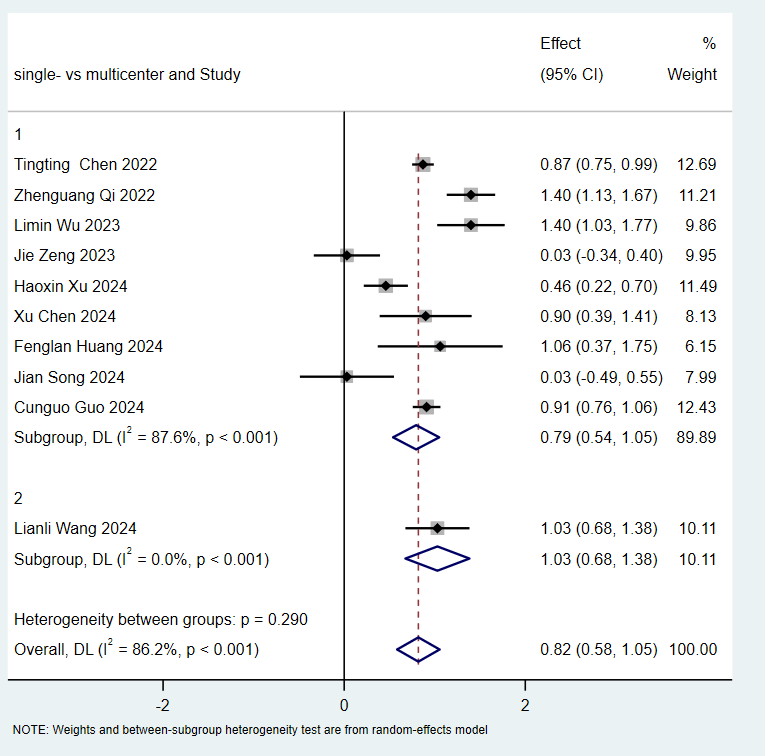  D |
| Subgroup analysis plots .(A)linaclotide treatment duration; (B)PEG dose; (C)split- vs. single-dose;(D)single- vs multicenter | |

| 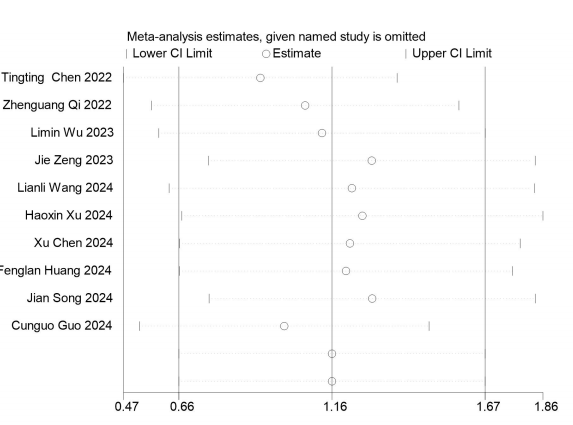A | 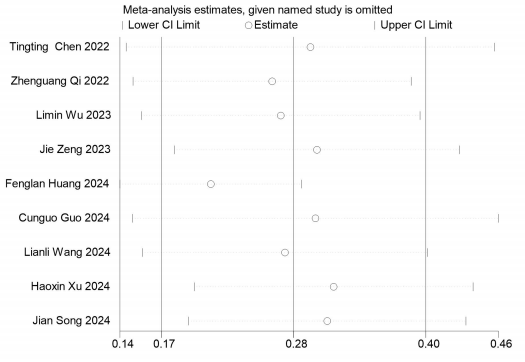B |
| --- | --- |
| 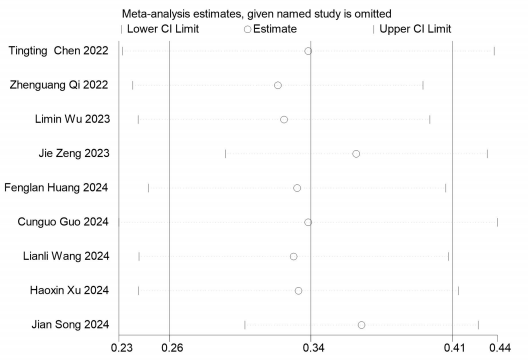C | 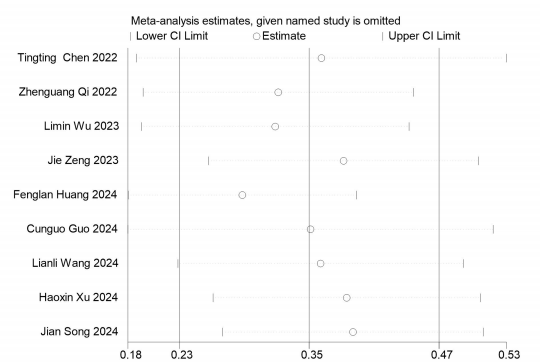D |
| Sensitivity analysis plots of BBPS.(A)Total Score; (B)Left colon Score; (C)Right colon Score;(D)Mid-colon Score | |
| 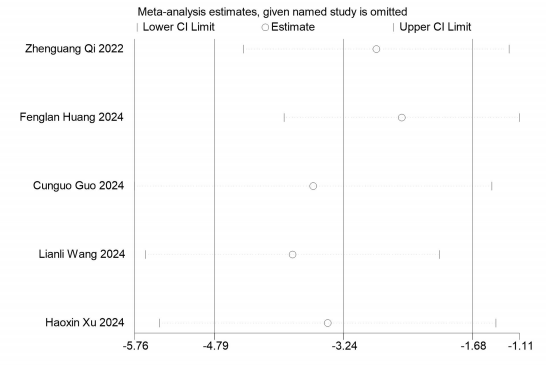E | 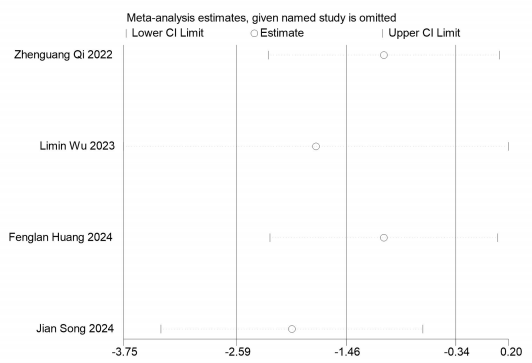F |
| Sensitivity analysis plots of total examination time | Sensitivity analysis plots of insert time |

2.Sensitivity analysis

3.Publication bias

| A | B |
| --- | --- |
| Funnel plots of BBPS total score.(A)Egger’s test;(B)Begg’s test | |
